# Supplementary material for: Hybrid Models and Biological Model Reduction with PyDSTool
Source: PLoS Comput Biol. 2012 Aug 9;8(8):e1002628. doi: 10.1371/journal.pcbi.1002628 (PMC3415397; doi:10.1371/journal.pcbi.1002628)
Supplement: Text S4 — Complete source code for the PyDSTool package (version 0.88.120504). Includes API documentation and help files linking to web pages. This file is identical to the current public release on Sourceforge.net. (ZIP) [file pcbi.1002628.s004.zip › PyDSTool/html/PyDSTool.ModelConstructor'-module.html]

xml version="1.0" encoding="ascii"?


PyDSTool.ModelConstructor'


| Home | Trees | Indices | Help | PyDSTool |
| --- | --- | --- | --- | --- |

Package PyDSTool ::
Module ModelConstructor'

[hide private]

[frames] | no frames]

# Module ModelConstructor'

source code

```
Model Constructor classes.

   Instantiate abstract Model Specifications into concrete simulation code.

   Robert Clewley, September 2005.


Overview of steps that ModelConstructor takes:

1. Build Generators for each vector field
 * Take flattened spec and select a compatible Generator type and language
   * include check that compatibleGen selection actually exists,
      and is compatible with specType/domain, and with cts/discrete
      time variables)
   * presently, each v.f. must have all the same Gen type
 * Map things like x^y and x**y power specs -> pow(x,y)
    and abs() function -> fabs() in C.

2. Given the specific target lang/Generator with a ModelSpec vector field
 * Associate additional events, code inserts, reused vars
 * Choose which domains map to domain verification events (certainly none that
    are infinite -- although semi-infinite can be checked in one direction)
 * All non-infinite domain ends would have an event created for them, but
    default is for them to be inactive.

 The 'mspec' argument to the GeneratorConstructor class must be a complete
 ModelSpec (up to introduction of global references including definitions of
 external inputs).

 The resulting model output from getModel contains the ModelSpec mspec in
 order to use its structure in resolving information about the relationship
 between variables.
```


|  |  |
| --- | --- |
| Classes | [hide private] |

Descriptor  
Abstract class for model and generator descriptors

EvMapping  
Event mapping class, for use by makeModelInfoEntry and, when
instantiated, the Model class.

GDescriptor  
All-in-one descriptor class for single Generators, and information
necessary to be able to build a Model object using a
ModelConstructor call -- i.e.

GeneratorConstructor

MDescriptor  
All-in-one descriptor class for hybrid model definitions and
information necessary to be able to build a Model object using a
ModelConstructor call.

ModelConstructor


|  |  |
| --- | --- |
| Functions | [hide private] |

|  |  |
| --- | --- |
| embed(gen, icdict=None, name=None, tdata=None, make\_copy=True)  Only use this function for building non-hybrid models with single Generators. | source code |

|  |  |
| --- | --- |
| makeEvMapping(mappingDict, varnames, parnames) | source code |

|  |  |
| --- | --- |
| makeModelInfo(arg) | source code |

|  |  |
| --- | --- |
| makeModelInfoEntry(dsi, allModelNames=None, swmap\_list=None, globcon\_list=None, nonevent\_reasons=None)  Create an entry for the modelInfo attribute of a Model or Generator, already wrapped in a dsInterface object. | source code |

|  |  |
| --- | --- |
| processReused(sourcenames, auxvarnames, flatspec, registry, FScompatibleNames, FScompatibleNamesInv)  Find and process reused terms in abstract specification. | source code |

|  |  |
| --- | --- |
| validateTransitionName(name, special\_reasons) | source code |


|  |  |
| --- | --- |
| Variables | [hide private] |

API = `API_class()`

Continuous = `Continuous Domain`

Discrete = `Discrete Domain`

Inf = `inf`

LargestInt32 = `2147483647`

NAMESEP = `'.'`

NaN = `nan`

\_all\_complex = `(<type 'complex'>, <type 'numpy.complexfloating...`

\_all\_float = `(<type 'float'>, <type 'numpy.floating'>, <type '...`

\_all\_int = `(<type 'int'>, <type 'numpy.integer'>, <type 'numpy...`

\_all\_numpy\_complex = `(<type 'numpy.complex128'>, <type 'numpy....`

\_all\_numpy\_float = `(<type 'numpy.float64'>, <type 'numpy.float...`

\_all\_numpy\_int = `(<type 'numpy.int32'>, <type 'numpy.int32'>, ...`

\_complex\_types = `(<type 'complex'>, <type 'numpy.complexfloati...`

\_float\_types = `(<type 'float'>, <type 'numpy.floating'>)`

\_int\_types = `(<type 'int'>, <type 'numpy.integer'>)`

\_num\_equivtype = `{<type 'float'>: <type 'numpy.float64'>, <typ...`

\_num\_maxmin = `{<type 'numpy.int32'>: [-2147483648, 2147483647]...`

\_num\_name2equivtypes = `{'float': (<type 'float'>, <type 'numpy...`

\_num\_name2type = `{'float': <type 'numpy.float64'>, 'int': <typ...`

\_num\_type2name = `{<type 'float'>: 'float', <type 'int'>: 'int'...`

\_num\_types = `(<type 'float'>, <type 'int'>, <type 'numpy.float...`

\_pytypefromtype = `{<type 'numpy.int32'>: <type 'int'>, <type '...`

\_real\_types = `(<type 'int'>, <type 'numpy.integer'>, <type 'fl...`

\_seq\_types = `(<type 'list'>, <type 'tuple'>, <type 'numpy.ndar...`

isfinite = `<ufunc 'isfinite'>`

mathNameMap = `{'Abs': 'abs', 'Acos': 'acos', 'Asin': 'asin', '...`

null\_predicate = `null_predicate_class(None)`

targetLangs = `['c', 'python', 'matlab']`


|  |  |
| --- | --- |
| Function Details | [hide private] |

|  |  |
| --- | --- |
| embed(gen, icdict=None, name=None, tdata=None, make\_copy=True) | source code |

Only use this function for building non-hybrid models with single
Generators. Otherwise, use the ModelConstructor class.

NB The supplied Generator is \*copied\* into the model unless optional
make\_copy argument is False.

|  |  |
| --- | --- |
| makeModelInfoEntry(dsi, allModelNames=None, swmap\_list=None, globcon\_list=None, nonevent\_reasons=None) | source code |

Create an entry for the modelInfo attribute of a Model or Generator,
already wrapped in a dsInterface object. Specify the list of non-event
based reasons which can be generated by global consistency checks.

|  |  |
| --- | --- |
| processReused(sourcenames, auxvarnames, flatspec, registry, FScompatibleNames, FScompatibleNamesInv) | source code |

Find and process reused terms in abstract specification. To avoid RHS
specs depending on auxiliary variables, temp variables will be declared
in FuncSpec.py and used in both the RHS and auxiliary variables in the
target language specification.

  


|  |  |
| --- | --- |
| Variables Details | [hide private] |

### \_all\_complex

Value:
:   ```
    (<type 'complex'>,
     <type 'numpy.complexfloating'>,
     <type 'numpy.complex128'>,
     <type 'numpy.complex64'>,
     <type 'numpy.complex128'>)
    ```

### \_all\_float

Value:
:   ```
    (<type 'float'>,
     <type 'numpy.floating'>,
     <type 'numpy.float64'>,
     <type 'numpy.float32'>,
     <type 'numpy.float64'>)
    ```

### \_all\_int

Value:
:   ```
    (<type 'int'>,
     <type 'numpy.integer'>,
     <type 'numpy.int32'>,
     <type 'numpy.int32'>,
     <type 'numpy.int8'>,
     <type 'numpy.int16'>,
     <type 'numpy.int32'>,
     <type 'numpy.int64'>)
    ```

### \_all\_numpy\_complex

Value:
:   ```
    (<type 'numpy.complex128'>,
     <type 'numpy.complex64'>,
     <type 'numpy.complex128'>)
    ```

### \_all\_numpy\_float

Value:
:   ```
    (<type 'numpy.float64'>,
     <type 'numpy.float32'>,
     <type 'numpy.float64'>)
    ```

### \_all\_numpy\_int

Value:
:   ```
    (<type 'numpy.int32'>,
     <type 'numpy.int32'>,
     <type 'numpy.int8'>,
     <type 'numpy.int16'>,
     <type 'numpy.int32'>,
     <type 'numpy.int64'>)
    ```

### \_complex\_types

Value:
:   ```
    (<type 'complex'>, <type 'numpy.complexfloating'>)
    ```

### \_num\_equivtype

Value:
:   ```
    {<type 'float'>: <type 'numpy.float64'>,
     <type 'int'>: <type 'numpy.int32'>,
     <type 'numpy.integer'>: <type 'numpy.int32'>,
     <type 'numpy.floating'>: <type 'numpy.float64'>,
     <type 'numpy.int8'>: <type 'numpy.int32'>,
     <type 'numpy.int16'>: <type 'numpy.int32'>,
     <type 'numpy.int32'>: <type 'numpy.int32'>,
     <type 'numpy.int32'>: <type 'numpy.int32'>,
    ...
    ```

### \_num\_maxmin

Value:
:   ```
    {<type 'numpy.int32'>: [-2147483648, 2147483647],
     <type 'numpy.float64'>: [-inf, inf]}
    ```

### \_num\_name2equivtypes

Value:
:   ```
    {'float': (<type 'float'>,
               <type 'numpy.floating'>,
               <type 'numpy.float64'>,
               <type 'numpy.float32'>,
               <type 'numpy.float64'>),
     'int': (<type 'int'>,
             <type 'numpy.integer'>,
             <type 'numpy.int32'>,
    ...
    ```

### \_num\_name2type

Value:
:   ```
    {'float': <type 'numpy.float64'>, 'int': <type 'numpy.int32'>}
    ```

### \_num\_type2name

Value:
:   ```
    {<type 'float'>: 'float',
     <type 'int'>: 'int',
     <type 'numpy.integer'>: 'int',
     <type 'numpy.floating'>: 'float',
     <type 'numpy.int8'>: 'int',
     <type 'numpy.int16'>: 'int',
     <type 'numpy.int32'>: 'int',
     <type 'numpy.int32'>: 'int',
    ...
    ```

### \_num\_types

Value:
:   ```
    (<type 'float'>,
     <type 'int'>,
     <type 'numpy.floating'>,
     <type 'numpy.integer'>)
    ```

### \_pytypefromtype

Value:
:   ```
    {<type 'numpy.int32'>: <type 'int'>,
     <type 'numpy.float64'>: <type 'float'>}
    ```

### \_real\_types

Value:
:   ```
    (<type 'int'>,
     <type 'numpy.integer'>,
     <type 'float'>,
     <type 'numpy.floating'>)
    ```

### \_seq\_types

Value:
:   ```
    (<type 'list'>, <type 'tuple'>, <type 'numpy.ndarray'>)
    ```

### mathNameMap

Value:
:   ```
    {'Abs': 'abs',
     'Acos': 'acos',
     'Asin': 'asin',
     'Atan': 'atan',
     'Atan2': 'atan2',
     'Betavariate': 'betavariate',
     'Ceil': 'ceil',
     'Choice': 'choice',
    ...
    ```

  


| Home | Trees | Indices | Help | PyDSTool |
| --- | --- | --- | --- | --- |

|  |  |
| --- | --- |
| Generated by Epydoc 3.0.1 on Fri May 4 15:24:03 2012 | http://epydoc.sourceforge.net |
